# Editorial: Salinity and drought stress in plants: understanding physiological, biochemical and molecular responses

**DOI:** 10.3389/fpls.2023.1277859

**Published:** 2023-10-09

**Authors:** Muhammad Waseem, Pingwu Liu, Mehtab Muhammad Aslam

**Affiliations:** ^1^ Sanya Nanfan Research Institute of Hainan University, Sanya, China; ^2^ Sanya Nanfan Research Institute, Fang Zhiyuan Academician Team Innovation Center of Hainan Province, Sanya, China; ^3^ Key Laboratory of Tropical Horticultural Crop Quality Regulation, College of Horticulture, Hainan University, Haikou, Hainan, China; ^4^ School of Life Sciences and State Key Laboratory of Agrobiotechnology, The Chinese University of Hong Kong, Shatin, China; ^5^ College of Agriculture, Food and Natural Resources (CAFNR), Division of Plant Sciences & Technology, University of Missouri, Columbia, MO, United States

**Keywords:** drought stress, salinity stress, food security, stress tolerance, plant breeding, plant physiology, omics

## 1 Introduction

The foremost abiotic stresses, such as salinity and drought, are major limiting factors that hamper agricultural productivity worldwide (Waadt et al., 2022). Both of these stresses can induce several physiological, morphological, and metabolic changes through various mechanisms, ultimately impacting plant growth, development, and final productivity. The influence of salinity/drought stresses is further exacerbated by global climatic changes affecting the frequency and magnitude of occurrence of these stresses (Parihar et al., 2015). In the current scenario, the development of groundbreaking strategies for sustainable crop production is crucial in order to effectively mitigate the challenges presented by salinity and drought stresses (Rajput et al., 2021). Against this backdrop, significant progress has been made in decoding the key characteristics of salinity and drought stress, including their underlying physiological, biochemical, and molecular bases; such findings have greatly assisted in breeding of stress-tolerant crops (Kumar et al., 2023). However, our understanding of the accumulated knowledge regarding salinity/drought tolerance remains a persistent challenge (Zhang et al., 2022). The key challenges encompass (i) evolution of salinity/drought resistance, (ii) key genes in genetic and regulatory mechanisms at the transcriptional and posttranscriptional level, (iii) transfer of accumulated knowledge from laboratory to field for sustainable production, and (iv) biochemical alterations and metabolic adjustments to avoid cellular damage. To address these challenges, it is imperative to establish research initiatives that aim to target the combined effects of salinity and drought stresses. Such research should focus on exploration at the genetic, molecular, biochemical, and physiological levels for the development of sustainable agriculture in the era of climate change.

## 2 Genome-wide functional analysis

Genome-wide gene functional analysis is a valuable tool for identifying key stress-related genes, which can then be characterized to better understand how plants are adapted to salinity and drought. Osmotic stress tolerance is primarily mediated by abscisic acid (ABA), and plants achieve osmotic stress tolerance through both ABA-dependent and ABA-independent mechanisms. Recently, researchers have highlighted the role of WRKY transcription factors in enhancing salinity and drought tolerance in plants through ABA-mediated regulation. For instance, in Kenaf (*Hibiscus cannabinus* L.), Chen et al. report that *HcWRKY44*, among 46 WRKY genes, acts as positive regulator of salinity tolerance through ABA-mediated regulation. Likewise, in *Iris germanica*, two WRKY genes (*IgWRKY50* and *IgWRKY32*) play a role in regulating the ABA signal transduction pathway and controlling stomatal aperture to improve drought tolerance (Zhang et al.). These studies offer a comprehensive understanding of WRKY genes, their potential in the development of plants with tolerance to salinity/drought stresses, and their utmost important in breeding and production of stress-tolerant crop varieties.

## 3 Integrated high-throughput approaches

Functional analysis relies on high-throughput techniques. For instance, Yao et al. performed transcriptome analysis to uncover the molecular mechanism behind leaf thickening under salinity stress in the halophytic plant *Lycium barbarum*. Their study identified a total of 3572 DEGs, among which expansin protein *EXLA2* acts as a positive regulator of palisade tissue thickness in *L. barbarum* leaves. In another study, Wu et al. uncovered the role of a glycolytic enzyme enolase 2 (ENO2) in seed germination under salt stress in Arabidopsis. They integrated transcriptomic and TMT-based proteomic approaches, revealing an array of salt stress-responsive genes in *AtENO2* mutant (*eno2* ^-^) and wild-type Arabidopsis. Their study shows that GAPA1/GAPB interacts with *AtENO2* to regulate seed germination under salinity stress, which could be leveraged to enhance plant tolerance to saline environments.

Water scarcity can limit soil nutrient solubilization and mineralization, affecting nutrient kinetics at the soil–root interface and resulting in impairments to leaf morphology and reduced stomatal conductance. To address this challenge, screening of agricultural crop germplasm could be conducted using integrated high-throughput techniques in order to develop crops with desirable traits. Kim et al. conducted a study on the quality and nutrition of tomato fruit under water stress with varying nutrient status. By using integrated GC-TOF-MS and UHPLC-LTQ-Orbitrap-MS approaches, they found that water stress shifted the primary metabolites of tomato fruits in different ways in soils with different nutrient conditions. On this basis, considering the nutritional quality of tomato fruit (particularly in relation to sugars and amino acids), the authors do not recommend propagation of tomatoes in water-deficient soils with surplus nutrients.

Shifting to another context, *Auricularia fibrillifera*, the third most widely cultivated mushroom and a common traditional Chinese food and medicine enriched in melanin, folate, and biotin, was studied by Guo et al. under desiccation stress. The focus was on transcriptomes and proteomes of fruit bodies under exposure to desiccation stress, rehydration, and regular watering. The research revealed that proteomics data aligned with transcriptomic data, particularly in pathways related to antibiotic biosynthesis, biosynthesis of biotin and folate, and amnio acid biosynthesis. This study provides valuable insights into desiccation tolerance in *A. fibrillifera.*

## 4 ncRNAs as emerging regulators of salinity/drought stress tolerance

Non-coding RNAs (ncRNAs) are emerging regulators of various biological processes, including stress tolerance (Waseem et al., 2021; Waseem et al., 2022; Aslam et., 2022). Zou et al. report 32017 long non-coding RNAs (lncRNAs) in *Beta vulgaris*, revealing 386 differentially expressed lncRNAs, including two significantly expressed lncRNAs (TCONS_00038334/TCONS_00055787) in response to drought. They also identified 42 lncRNAs as potential miRNA target mimics. In another study, Ji et al. showed that that the miR169b/NFYA1 module in the halophyte *Halostachys capsica* enhances salinity and drought tolerance by enhancing ABA biosynthesis, modulating ABA signal transduction pathways, and maintaining ROS homeostasis.

## 5 Exploration of underlying salinity/drought tolerance mechanisms

In the current era of global climatic change, the relationship between climate and crop yield is evolving, especially for global food security crops such as wheat. Drought directly impacts wheat production. Ghaffar et al. conducted a study evaluating agronomic adaptions for drought tolerance using Pakistan’s wheat germplasm. They investigated 40 local wheat cultivars for various properties, including growth, pigmentation, and ROS accumulation capacity. Ghaffar et al. successfully identified drought-tolerant (Barani-83/Blue silver) and drought-sensitive (FSD-08/Lasani-08) cultivars. Another approach to enhancing drought stress in winter wheat involves foliar application of Zinc (Zn). Zarea and Karimi show that combing the foliar application of Zn with 6-Benzylaminopurine (6-BAP) promotes growth and improves drought tolerance, preventing grain yield reduction in winter wheat.

We appreciate the contribution by Schierenbeck et al., who conducted a quantitative trait nucleotide (QTN) analysis of a diverse winter wheat panel (261 accessions) under drought stress. The authors successfully identified a QTN, TraesCS2D02G133900, that may play a role in controlling shoot and coleoptile length. This study highlights the significance of genetics factors in response to drought in wheat, offering insights for wheat adaptation and improvement.

Ran et al. performed a comparative analysis of salinity tolerance in three willow species, *Salix matsudana*, *S. gordejevii*, and *S. linearistipularis.* Their study revealed that *S. linearistipularis* is salt-sensitive and could be planted as an indicative plant for salinity. Meanwhile, Zhang et al. identified the formation of accessory structures, in the form of salt bladders, in *Chenopodium album.* These structures serve as an adaptive measure to prevent salt sensitivity in this plant. Additionally, Chandrasekaran et al. found that, under drought stress, *Pinus strobus* shifts carbon partitioning toward biosynthesis of volatile organic compounds (VOCs), particularly monoterpene synthesis. Lastly, Hussain et al. demonstrated that selenium seed priming in *Brassica rapa* mitigates salinity stress by activating stress-responsive genes and improving the ROS system.

On the basis of this comprehensive collection of research on the topic at hand, various invaluable insights into salinity and drought resistance have accumulated. We hope that this accumulated knowledge will assist with and pave the path for new studies and breeding programs aiming to develop salinity- and drought-resilient crops. To achieve a better understanding of salinity and drought tolerance in plants, a holistic approach involving integrated variety improvement, theoretical research, and field management is required. We sincerely appreciate the efforts and significant contributions of the authors, editors, and peer reviewers whose efforts and significant contributions have made this Research Topic possible. We hope that our readers will uncover valuable information within this Research Topic collection and identify potential collaborators in this critical area.

## Author contributions

MW: Conceptualization, Supervision, Validation, Writing – original draft. PL: Writing – review & editing. MMA: Writing – review & editing.
